# Supplementary figures and images for: Egg-adaptive mutations of human influenza H3N2 virus are contingent on natural evolution
Source: PLoS Pathog. 2022 Sep 26;18(9):e1010875. doi: 10.1371/journal.ppat.1010875 (PMC9536752; doi:10.1371/journal.ppat.1010875)

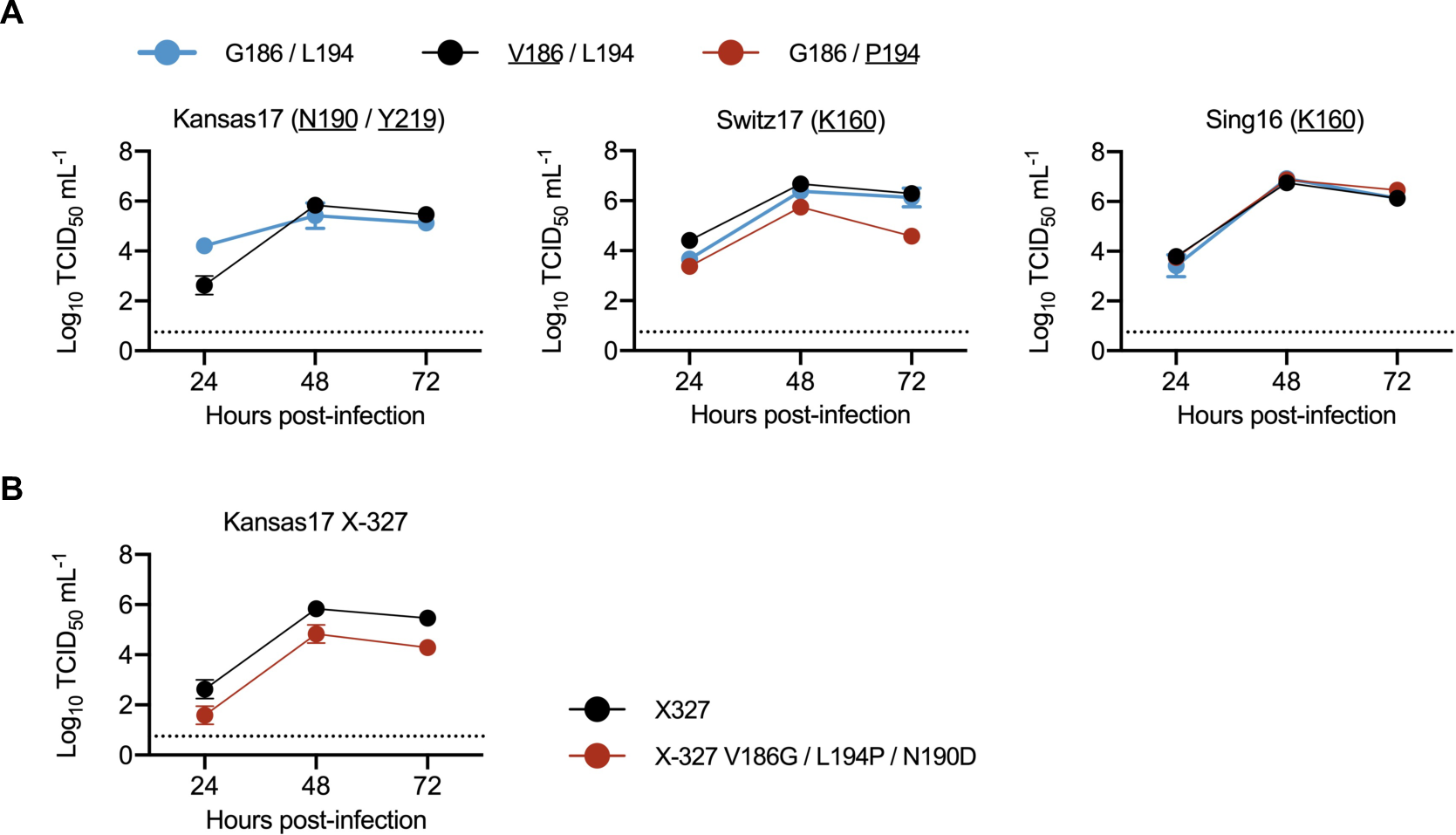

Supplement: S1 Fig — (A) Viral replication kinetics of rescuable mutants of H3N2 vaccine strains were examined in hMDCK cells. (B) Viral replication kinetics of Kansas17 X-327 and Kansas17 X-327 with V186G/L194P/N190D were examined in hMDCK cells. (A, B) hMDCK cells were inoculated with the indicated variant at an MOI of 0.01. Viral titers in supernatants harvested at 24, 48 and 72 h post-infection were measured by TCID50 using hMDCK cells. The means of three independent experiments are shown with SD indicated by the error bars. The dashed line represents the lower detection limit. Amino acid variant representing an egg-adaptive mutation is underlined. (TIF) [file ppat.1010875.s001.tif]
