# Supplementary material for: Egg-adaptive mutations of human influenza H3N2 virus are contingent on natural evolution
Source: PLoS Pathog. 2022 Sep 26;18(9):e1010875. doi: 10.1371/journal.ppat.1010875 (PMC9536752; doi:10.1371/journal.ppat.1010875)
Supplement: S1 Table — (DOCX) [file ppat.1010875.s002.docx]

**S1 Table. H3N2 components of WHO-recommended influenza vaccines for influenza seasons between 2008 and 2023 [1].**

| **SEASON** | **STRAIN NAME** |
| --- | --- |
| 2008-2010 | A/Brisbane/10/2007 (IVR-147, X-171) |
| 2010-2012 | A/Perth/16/2009 (NIB-64) |
| 2012-2014 (Northern hemisphere) | A/Victoria/361/2011 (NYMC-X-217, IVR-165) |
| 2013 (Southern hemisphere) |  |
| 2014 (Southern hemisphere) | A/Texas/50/2012 (X-223) |
| 2014-2015 (Northern hemisphere) |  |
| 2015 (Southern hemisphere) | A/Switzerland/9715293/2013 (NIB-88, NYMC-X-247, IVR-176) |
| 2015-2016 (Northern hemisphere) |  |
| 2016-2017 (Southern hemisphere) | A/Hong Kong/4801/2014 (X-263) |
| 2016-2018 (Northern hemisphere) |  |
| 2018 (Southern hemisphere) | A/Singapore/INFIMH-16-0019/2016  (IVR-186, NYMC-X-307, NIB-104) |
| 2018-2019 (Northern hemisphere) |  |
| 2019 (Southern hemisphere) | A/Switzerland/8060/2017 (NIB-112) |
| 2019-2020 (Northern hemisphere) | A/Kansas/14/2017 (NYMC-X-327, IVR-195) |
| 2020 (Southern hemisphere) | A/South Australia/34/2019 (IVR-197) |
| 2020-2021 (Northern hemisphere) | A/Hong Kong/2671/2019 (NIB-121) |
| 2021 (Southern hemisphere) |  |
| 2021-2022 (Northern hemisphere) | A/Cambodia/e0826360/2020 (NYMC-X-361, IVR-224) |
| 2022 (Southern hemisphere) | A/Darwin/9/2021 (IVR-228, NYMC-X-369) |
| 2022-2023 (Northern hemisphere) |  |

**REFERENCES**

1. World Health Organization. Recommendations for influenza vaccine composition [cited 2022]. Available from: <https://www.who.int/teams/global-influenza-programme/vaccines/who-recommendations>.
