# Supplementary material for: Egg-adaptive mutations of human influenza H3N2 virus are contingent on natural evolution
Source: PLoS Pathog. 2022 Sep 26;18(9):e1010875. doi: 10.1371/journal.ppat.1010875 (PMC9536752; doi:10.1371/journal.ppat.1010875)
Supplement: S2 Table — (DOCX) [file ppat.1010875.s003.docx]

**S2 Table. H3N2 genes that were used in this study.**

| **PARENTAL VIRUS** | **STRAIN NAME** | **CLADE** | **GENE** | **EGG-ADAPTIVE MUTATION** | **GISAID ACCESSION NO.** |
| --- | --- | --- | --- | --- | --- |
| A/Kansas/14/2017 | X-327 | 3C.3a1 | HA | G186V, D190N, S219Y | EPI1490170 |
|  |  |  | NA | - | EPI1504534 |
| A/Switzerland/8060/2017 | NIB-112 | 3C.2a2 | HA | T160K, L194P | EPI1313524 |
|  |  |  | NA | - | EPI1313525 |
| A/Singapore/INFIMH-16-0019/2016 | WT | 3C.2a1 | HA | - | EPI1381186 |
|  |  |  | NA | - | EPI1381185 |
| A/Italy/11871/2020 | WT | 3C.2a1b.1a | HA | - | EPI1735610 |
|  |  |  | NA | - | EPI1735609 |
| A/Victoria/22/2020 | WT | 3C.2a1b.1a | HA | - | EPI1721444 |
|  |  |  | NA | - | EPI1721443 |

‘-‘: no egg-adaptive mutation.
