# Supplementary material for: Egg-adaptive mutations of human influenza H3N2 virus are contingent on natural evolution
Source: PLoS Pathog. 2022 Sep 26;18(9):e1010875. doi: 10.1371/journal.ppat.1010875 (PMC9536752; doi:10.1371/journal.ppat.1010875)
Supplement: S3 Table — (DOCX) [file ppat.1010875.s004.docx]

**S3 Table. Frequencies of mutations in egg-passaged viruses that showed an average frequency of >10% in the fifth passage.**

| **INOCULUM** | **MUTATION** | **BIOLOGICAL REPLICATE** | **FREQUENCY DURING EGG-PASSAGING** | | | | |
| --- | --- | --- | --- | --- | --- | --- | --- |
|  |  |  | **E1** | **E2** | **E3** | **E4** | **E5** |
| Kansas17  V186/N190/L194/Y219  (X-327) | - | Rep1 | - | - | - | - | - |
|  |  | Rep2 | - | - | - | - | - |
|  |  | Rep3 | - | - | - | - | - |
| Switz17  K160/G186/P194 | T203I | Rep1 | 0.01 | 0.62 | 0.97 | 0.98 | 0.98 |
|  |  | Rep2 | 0.06 | 0.14 | 0.32 | 0.53 | 0.85 |
|  |  | Rep3 | 0.08 | 0.13 | 0.23 | 0.57 | 0.75 |
| Sing16  K160/G186/P194 | I140K | Rep1 | 0.00 | 0.00 | 0.00 | 0.00 | 0.00 |
|  |  | Rep2 | 0.00 | 0.03 | 0.49 | 0.48 | 0.73 |
|  |  | Rep3 | 0.00 | 0.00 | 0.00 | 0.00 | 0.00 |
|  | T203I | Rep1 | 0.01 | 0.01 | 0.07 | 0.21 | 0.27 |
|  |  | Rep2 | 0.45 | 0.16 | 0.43 | 0.49 | 0.24 |
|  |  | Rep3 | 0.01 | 0.01 | 0.02 | 0.57 | 0.90 |
| Kansas17 X-327  V186G/L194P/N190D | I140K | Rep1 | 0.00 | 0.00 | 0.00 | 0.02 | 0.06 |
|  |  | Rep2 | 0.15 | 0.44 | 0.71 | 0.82 | 0.87 |
|  |  | Rep3 | 0.02 | 0.05 | 0.14 | 0.19 | 0.28 |
|  | T203I | Rep1 | 0.37 | 0.70 | 0.88 | 0.86 | 0.93 |
|  |  | Rep2 | 0.00 | 0.01 | 0.01 | 0.01 | 0.00 |
|  |  | Rep3 | 0.03 | 0.07 | 0.17 | 0.22 | 0.21 |
|  | D225N | Rep1 | 0.00 | 0.00 | 0.02 | 0.10 | 0.28 |
|  |  | Rep2 | 0.01 | 0.02 | 0.02 | 0.01 | 0.01 |
|  |  | Rep3 | 0.02 | 0.10 | 0.25 | 0.34 | 0.39 |

Continued

| **INOCULUM** | **MUTATION** | **BIOLOGICAL REPLICATE** | **FREQUENCY DURING EGG-PASSAGING** | | | | |
| --- | --- | --- | --- | --- | --- | --- | --- |
|  |  |  | **E1** | **E2** | **E3** | **E4** | **E5** |
| Switz17  K160/V186/L194 | - | Rep1 | - | N/A | N/A | N/A | N/A |
|  |  | Rep2 | - | N/A | N/A | N/A | N/A |
|  |  | Rep3 | - | N/A | N/A | N/A | N/A |
| Sing16  K160/V186/L194 | H156R | Rep1 | 0.00 | 0.00 | N/A | N/A | N/A |
|  |  | Rep2 | 0.00 | 0.98 | N/A | N/A | N/A |
|  |  | Rep3 | 0.00 | 0.02 | N/A | N/A | N/A |
|  | D190N | Rep1 | 0.00 | 0.00 | N/A | N/A | N/A |
|  |  | Rep2 | 0.00 | 0.00 | N/A | N/A | N/A |
|  |  | Rep3 | 0.00 | 0.94 | N/A | N/A | N/A |
|  | D225N | Rep1 | 0.12 | 0.45 | N/A | N/A | N/A |
|  |  | Rep2 | 0.00 | 0.00 | N/A | N/A | N/A |
|  |  | Rep3 | 0.40 | 0.98 | N/A | N/A | N/A |

‘-’: no mutation found. N/A: the copy number of viral RNA was insufficient for deep sequencing analysis.
