# Supplementary material for: Egg-adaptive mutations of human influenza H3N2 virus are contingent on natural evolution
Source: PLoS Pathog. 2022 Sep 26;18(9):e1010875. doi: 10.1371/journal.ppat.1010875 (PMC9536752; doi:10.1371/journal.ppat.1010875)
Supplement: S4 Table — (DOCX) [file ppat.1010875.s005.docx]

**S4 Table. Information of HA from human H3N2 strains with or without egg-passaging.**

| **VIRUS NAME** | **NON-EGG-PASSAGED** | | **EGG-PASSAGED** | | **MUTATION IN EGG-PASSAGED** | | | | | |
| --- | --- | --- | --- | --- | --- | --- | --- | --- | --- | --- |
|  | **PASSAGE HISTORY** | **HA ACCESSION NO.** | **PASSAGE HISTORY** | **HA ACCESSION NO.** | **160** | **186** | **190** | **194** | **219** | **225** |
| A/Bangladesh/3005/2020 | Original | EPI1838357 | E3 | EPI1844062 | I | N | N | L | S | G |
|  |  |  | E3+E6 | EPI1857491 | I | N | N | L | S | G |
| A/Bangladesh/3011/2020 | Original | EPI1838349 | E3 | EPI1844060 | I | N | N | L | S | G |
| A/Bangladesh/4002/2020 | Original | EPI1838416 | E3 | EPI1844058 | I | N | N | L | S | G |
| A/Bangladesh/911009/2020 | Original | EPI1838408 | E3+E6 | EPI1857489 | I | N | N | L | S | G |
| A/Beijing-Daxin/33/2020 | C2 | EPI1753671 | E7 | EPI1753663 | K | D | N | L | S | D |
| A/Beijing-Miyun/51/2020 | C1 | EPI1753655 | E7+E2 | EPI1805765 | A | D | N | L | Y | G |
|  |  |  | E9 | EPI1801276 | I | D | N | L | F | G |
| A/Beijing-Miyun/53/2020 | C1 | EPI1753639 | E5+E2 | EPI1806767 | I | D | N | L | F | G |
| A/Beijing-Miyun/54/2020 | C1 | EPI1753623 | E4 | EPI1753615 | T | V | N | L | S | G |
| A/Belgium/G0023/2019 | SIAT1 | EPI1436935 | E3 | EPI1592043 | K | G | D | L | S | G |
| A/Brisbane/148/2019 | SIAT1+SIAT1 | EPI1658656 | E5 | EPI1740888 | K | V | D | L | Y | D |
| A/Brunei/39/2020 | SIAT1 | EPI1797893 | E5 | EPI1804363 | K | D | N | L | S | D |
| A/California/194/2019 | Original | EPI1630686 | E5 | EPI1713739 | K | V | D | L | S | D |
| A/Cambodia/e0826360/2020 | Original | EPI1837753 | E5+E1+E8 | EPI1877927 | K | R | D | L | F | D |
| A/Canberra/407/2019 | SIAT1 | EPI1671923 | E4 | EPI1713737 | K | G | D | P | S | D |
| A/Christchurch/515/2019 | SIAT2 | EPI1484393 | E3+E2 | .2a1b.2b | T | G | D | P | F | D |
| A/Christchurch/516/2019 | SIAT1 | EPI1484453 | E3 | EPI1491158 | K | V | D | L | F | D |
| A/Darwin/1/2021 | Original | EPI1851811 | E8 | EPI1859990 | K | S | D | L | S | G |
| A/Darwin/11/2021 | Original | EPI1859986 | E3 | EPI1859998 | I | D | N | L | S | D |
| A/Darwin/17/2021 | SIAT1 | EPI1888120 | E3 | EPI1924410 | I | V | N | L | S | D |
| A/Darwin/2/2021 | Original | EPI1851819 | E3 | EPI1859970 | K | R | D | L | S | D |
| A/Darwin/22/2021 | SIAT1 | EPI1888104 | E3 | EPI1923164 | I | N | N | L | S | G |

Continued

| **VIRUS NAME** | **NON-EGG-PASSAGED** | | **EGG-PASSAGED** | | **MUTATION IN EGG-PASSAGED** | | | | | |
| --- | --- | --- | --- | --- | --- | --- | --- | --- | --- | --- |
|  | **PASSAGE HISTORY** | **HA ACCESSION NO.** | **PASSAGE HISTORY** | **HA ACCESSION NO.** | **160** | **186** | **190** | **194** | **219** | **225** |
| A/Darwin/24/2021 | Original | EPI1888096 | E3 | EPI1923174 | I | N | N | L | S | G |
| A/Darwin/402/2019 | SIAT1 | EPI1508629 | E3 | EPI1584616 | T | G | D | P | F | N |
| A/Darwin/6/2021 | Original | EPI1857216 | E3+E7+E1 | EPI1885098 | I | N | N | L | S | G |
|  |  |  | E5+E2 | EPI1925255 | I | D | N | L | F | G |
| A/Darwin/726/2019 | SIAT1 | EPI1658695 | E6 | EPI1675460 | A | V | E | L | S | D |
| A/Darwin/9/2021 | SIAT1 | EPI1883349 | E4 | EPI1888006 | I | N | N | L | S | G |
| A/Delaware/01/2021 | Original | EPI1869534 | E2 | EPI1940656 | I | D | N | L | S | G |
| A/Finland/183/2020 | SIAT1 | EPI1753460 | E6 | EPI1847912 | K | S | D | L | S | D |
| A/Hong Kong/2671/2019 | MDCK1 | EPI1543098 | E9 | EPI1843071 | I | V | D | L | F | N |
| A/Kansas/14/2017 | SIAT2 | EPI1504535 | E17 | EPI1415371 | K | V | N | L | Y | D |
| A/KANAGAWA/ZC1841/2019 | SIAT1 | EPI1478189 | E7 | EPI1696421 | K | V | D | L | S | D |
| A/Michigan/173/2020 | Original | EPI1843859 | E4+E2 | EPI1922037 | I | N | N | L | S | G |
| A/Netherlands/00007/2021 | Original | EPI1885138 | E3 | EPI1924781 | I | N | N | L | S | G |
| A/Newcastle/42/2019 | SIAT1 | EPI1430423 | E3 | EPI1444940 | K | V | D | L | F | D |
| A/Newcastle/623/2019 | SIAT1 | EPI1430438 | E2 | EPI1444941 | K | G | D | L | S | G |
| A/Norway/16606/2021 | SIAT1 | EPI1922181 | E3 | EPI1940648 | I | N | N | L | S | G |
| A/Norway/2279/2019 | SIAT1 | EPI1619464 | E4 | EPI1719268 | K | G | D | P | S | D |
| A/Oregon/28/2019 | Original | EPI1627961 | E6 | EPI1713733 | K | V | D | L | S | D |
| A/Paris/2554/2019 | Original | EPI1638885 | E4+E7 | EPI1794629 | I | D | N | L | S | G |
| A/Pennsylvania/01/2021 | Original | EPI1858654 | E5 | EPI1924787 | I | G | N | L | S | G |
|  |  |  | E4 | EPI1924789 | I | D | N | L | S | G |

Continued

| **VIRUS NAME** | **NON-EGG-PASSAGED** | | **EGG-PASSAGED** | | **MUTATION IN EGG-PASSAGED** | | | | | |
| --- | --- | --- | --- | --- | --- | --- | --- | --- | --- | --- |
|  | **PASSAGE HISTORY** | **HA ACCESSION NO.** | **PASSAGE HISTORY** | **HA ACCESSION NO.** | **160** | **186** | **190** | **194** | **219** | **225** |
| A/Pennsylvania/1025/2019 | Original | EPI1630907 | E3+E1 | EPI1796140 | I | V | D | L | S | N |
|  |  |  | E3+D8+E1 | EPI1804937 | I | V | E | L | S | D |
|  |  |  | E3 | EPI1713729 | I | V | D | L | S | D |
| A/Pennsylvania/1026/2019 | Original | EPI1631152 | E5+E8 | EPI1794631 | K | V | D | L | Y | D |
|  |  |  | E5+E2+E9 | EPI1843569 | K | V | D | L | Y | D |
| A/Perth/20/2020 | MDCK1+SIAT1 | EPI1733852 | E3+E2+E9 | EPI1848094 | K | D | N | L | Y | G |
|  |  |  | E3 | EPI1740884 | K | D | N | L | Y | D |
|  |  |  | E3+E7 | EPI1794633 | K | D | N | L | Y | N |
| A/Saitama/92/2020 | MDCK1+hMDCK1 | EPI1847848 | E4 | EPI1847862 | K | R | D | L | S | D |
| A/Singapore/INFKK0001/2021 | Original | EPI1883806 | E4 | EPI1889199 | I | N | N | L | S | G |
| A/Singapore/INFKK0002/2021 | Original | EPI1883814 | E4 | EPI1924397 | I | N | N | L | S | G |
| A/Singapore/KK0001/2020 | Original | EPI1750786 | E3 | EPI1804361 | K | D | D | L | S | D |
| A/South Africa/R06421/2019 | MDCK1+SIAT1 | EPI1582733 | E4 | EPI1694137 | K | V | D | L | F | D |
| A/South Australia/2/2019 | SIAT1 | EPI1387412 | E4+E2 | EPI1698481 | I | D | D | L | S | D |
| A/South Australia/320/2019 | SIAT1 | EPI1484436 | E4 | EPI1526498 | T | G | D | P | F | D |
| A/South Australia/34/2019 | SIAT1 | EPI1387331 | E5 | EPI1703041 | K | I | D | L | F | D |
| A/South Australia/36/2019 | SIAT1 | EPI1387334 | E4 | EPI1440496 | T | G | D | P | F | D |
| A/South Australia/39/2019 | SIAT1 | EPI1387337 | E4 | EPI1440498 | K | G | D | P | S | D |
| A/South Australia/4/2019 | SIAT1 | EPI1371913 | E4+E2 | EPI1698473 | K | D | D | L | S | D |
| A/Sydney/53/2019 | MDCK-SIAT1+SIAT1 | EPI1430420 | E3+E2 | EPI1588472 | K | V | D | L | F | D |
| A/Tasmania/503/2020 | SIAT1 | EPI1752480 | E5 | EPI1848147 | K | R | D | L | S | D |
|  |  |  | EX | EPI1868371 | K | R | E | L | F | D |
|  |  |  | EX | EPI1868373 | K | R | D | L | F | D |

Continued

| **VIRUS NAME** | **NON-EGG-PASSAGED** | | **EGG-PASSAGED** | | **MUTATION IN EGG-PASSAGED** | | | | | |
| --- | --- | --- | --- | --- | --- | --- | --- | --- | --- | --- |
|  | **PASSAGE HISTORY** | **HA ACCESSION NO.** | **PASSAGE HISTORY** | **HA ACCESSION NO.** | **160** | **186** | **190** | **194** | **219** | **225** |
| A/Vermont/11/2019 | Original | EPI1428265 | E2 | EPI1439232 | K | G | N | L | S | D |
| A/Vermont/14/2019 | Original | EPI1428013 | E2 | EPI1439224 | K | G | N | L | S | D |
| A/Vermont/25/2019 | Original | EPI1618933 | E3 | EPI1713735 | K | V | D | L | S | D |
| A/Victoria/223/2019 | SIAT1 | EPI1584570 | E3 | EPI1610406 | T | G | D | P | F | D |
| A/Victoria/703/2019 | SIAT1 | EPI1430441 | E2 | EPI1444936 | K | G | D | L | S | G |

For passage history, ‘C’: passaged in cells. ‘E’: passaged in eggs. The passage number is indicated as the suffix. ‘X’ indicates missing information. Egg-adaptive mutations are highlighted in yellow.
