# Supplementary material for: Egg-adaptive mutations of human influenza H3N2 virus are contingent on natural evolution
Source: PLoS Pathog. 2022 Sep 26;18(9):e1010875. doi: 10.1371/journal.ppat.1010875 (PMC9536752; doi:10.1371/journal.ppat.1010875)
Supplement: Attachment — Submitted filename: Liang_PLOS Pathg Response_V7_20220728.docx [file ppat.1010875.s006.docx]

**Response to Reviewers’ Comments**

**Part I - Summary**

Reviewer #1: Liang et al. complete a nice set of experiments analyzing the compatibility of different egg-adaptative HA substitutions in different H3N2 backgrounds. Wu and colleagues recently demonstrated that G186V and L194P substitutions in HA are incompatible, and the current study expands this to examine additional substitutions in other HA backgrounds. They first identify different egg-adaptive substitutions in different egg based H3N2 vaccine strains. They then study replicative fitness (or ability to rescue) viruses with different combinations of HA mutations. They ultimately identify epistatic interactions, including those involving key egg/antigenic substitutions such as T160K, that are in these vaccine strains, and conclude that different egg-adaptations are favored in different genetic backgrounds.

**Response:** Thank you for the positive comments.

Reviewer #2: In this manuscript by Liang et al., the authors sought out to understand how egg adaptations of H3N2 differ across distinct H3N2 viruses. The authors nicely show epistasis of key residues associated with egg mutations and how this relates to distinct H3N2 clades. The manuscript is very well written and the topic is important. The manuscript can be hard to follow at times, largely due to the massive number of mutations being studied. Simplification of the key residues and relationship between these as a panel somewhere in the paper would greatly benefit the presentation of this manuscript. Otherwise, all my comments are quite minor and easily addressable.

**Response:** Thank you for your constructive comments. In our revised manuscript, we have added a figure (Figure 1B) to summarize the relationship between different egg-adaptive mutations and strains of interest. In addition, we have simplified our variant naming scheme to improve the clarity of the manuscript. For example, “Kansas17-D190N/S219Y” is renamed as “Kansas17-N190/Y219”.

Reviewer #3: The authors assess how natural mutations in seasonal H3N2 virus impact on the adaptation of these same viruses when cultured in eggs. H3N2 viruses are frequently associated to a higher mismatch of current seasonal vaccines in comparison to group 1 and influenza B viruses. One important aspect to consider here is that most currently licensed influenza vaccines are manufactured in eggs. Therefore, it is of interest to understand if changes in antigenicity of H3N2 viruses when passaged in eggs are related to specific DNA/protein sequences of circulating H3N2 influenza viruses. The manuscript is well-written and conveys a clear message to the audience. There are some minor points that should be addressed before acceptance.

**Response:** Thank you for the supportive comments. All minor points from this reviewer have been addressed in the revised manuscript (see below).

Reviewer #4: In this manuscript Liang et al, perform a series of experiments to elucidate the trajectory and limitations of the mutation (amino acid variations) that arise in the hemagglutinin (HA) protein of H3N2 human seasonal viruses during egg-adaptation. In previous studies they identified the amino acid change L194P as an important egg-adaptation that often occurs during passaging of human H3N2 in eggs. Herein they identify specific amino acid changes that appear to be compensatory mutations that are generated in the presence of amino acid P194 in in HA. The authors also identify two naturally occurring amino acids (T160 and N190) that are appear to be incompatible with mutation P194, suggesting a potential structural restrictions on accommodating some of these residue variations. The results provide relevant information on egg-adaptations that impact vaccine development. Nonetheless, there are a few issues that need to be addressed to improve the clarity of the manuscript, in particular on how the authors are assessing and interpreting fitness.

**Response:** Thank you for the constructive comments. Our manuscript is revised accordingly (see below).

**Part II – Major Issues: Key Experiments Required for Acceptance**

Reviewer #1:

1. Overall, the study is very nice and the experiments are well control and the data are solid. There are some parts of the manuscript where I felt ‘a bit in the weeds’ trying to follow along with different combinations of substitutions, but the overall experimental question is important and the data support the idea that different egg adaptations are favored in different genetic backgrounds.

**Response:** Thank you for your suggestion. In our revised manuscript, we have added a figure (Figure 1B) to summarize the relationship between different egg-adaptive mutations and strains of interest. In addition, we have simplified our variant naming scheme to improve the clarity of the manuscript. For example, “Kansas17-D190N/S219Y” is renamed as “Kansas17-N190/Y219”.

2. The study could be strengthened if there was a better structural/mechanistic explanation for some of the findings. For example, Wu and colleagues showed earlier that RBS height explained the incompatibility of L194P and G186V. The authors of the current study show that is not the case in panel Fig 3F for incompatibility of N190 and P194, but additional structural experiments could address the mechanism further about this incompatibility.

**Response:** We agree that additional structural experiments will be informative for understanding the epistatic interaction between N190 and P194. Currently, we are attempting to determine several relevant crystal structures, which should provide a better mechanistic explanation of the incompatibility between N190 and P194. We are also performing deep mutational scanning experiments to systematically map mutations that are involved in the epistatic network. We believe that these experiments are outside of the scope of present study and hope to publish them in a separate manuscript.

3. The authors conclude that the specific types of egg-adaptations that arise is complicated and dependent on the evolving H3N2 strains. It would be useful to be able to predict specific egg-adaptations based on sequence—is this impossible at this point or could preliminary predictive models be made?

**Response:** Thank you for bringing up this idea, which is now described in the discussion section of the revised manuscript (lines 223-229):

“Besides L194P, many other egg-adaptive mutations, albeit at a lower prevalence, were observed in historical vaccine strains, such as H156Q, H156R, H183L and A196T [14, 25-27]. The sequence determinants that influence the preference of other egg-adaptive mutations are currently unclear. Comprehending the epistatic interactions that involve egg-adaptive mutations as well as the underlying mechanisms will be key to accurately predict mutations that arise during egg-adaptation.”

Reviewer #2: N/A

Reviewer #3: (No Response)

Reviewer #4:

- It is unclear how the authors assessed replication fitness of the rescued viruses. In the methods they mention that after transfection the viruses were harvested and inoculated into hMDCKs, and then the supernatant were harvested once CPE was observed. These supernatants (containing the viruses) were then titrated by a TCID50 assay. Hence, it seems that the authors are determining fitness as a measure ability to rescue viruses and the generation of progeny virus (titer) after CPE was observed.

Was there any standardization to control the time that each of the viral infections took to generate CPE?; e.g. Do you collect the supernatant at the first sign of CPE? After a few hours of CPE? Until all the cells show CPE?

**Response:** Thank you for your useful comments. Our protocol for virus rescue is clarified in the materials and methods of the revised manuscript (lines 298-301):

“Supernatant was harvested at 72 h post-transfection, all of which was further inoculated into hMDCK cells at 90% confluence in a T75 flask. Supernatant of the hMDCK cells was harvested when more than 70% of cells had cytopathic effect (CPE). If less than 70% of cells had CPE by 72 h post-infection, cell supernatant was harvested regardless.”

In the revised manuscript, we have also included an experiment to measure the viral replication kinetics, which should provide a more objective comparison among viral mutants (see response to the next comment).

- To determine differences in replication fitness, one would have to perform a side-by-side growth curve for 48 hrs and plot them as such to determine and compare the kinetics of replication. As this is a major methodology of the paper (shown in Figs 1B, 2B and E, and 3B-D), these experiments need to be explained in more detail to understand how the authors are controlling for time differences that the cultures take to determine CPE, and the time when the sups were collected. It will also be valuable to actually show growth curves in cases where the growth kinetics are in fact slower (or faster).

**Response:** Thank you for the suggestion. The results of a replication kinetics experiment are included in our revised manuscript as Supplementary Figure 1. The results are described in the results section:

Lines 121-123: “Furthermore, all rescuable variants showed comparable replication kinetics in hMDCK cells, despite G186/P194 had a slightly lower titer in Switz17 and V186/L194 had a slower growth in Kansas17 (Supplementary Figure 1A)”

Lines 148-151: “Interestingly, although Kansas17 X-327 with V186G/L194P/N190D had a lower replication fitness in hMDCK cells compared to X-327, it showed comparable replication fitness with X-327 in eggs (Figure 2C and Supplementary Figure 1B).”

Detailed methodology is provided in the materials and methods section of the revised manuscript (lines 310-314):

“**Multicycle viral growth kinetics**

To measure the viral multicycle viral growth kinetics, rescuable viruses were inoculated into hMDCK cells at an MOI of 0.01, and supernatants were harvested at 24, 48 and 72 h post-infection [44]. Viral titers in the supernatants were determined by titration in hMDCK cells as previously described [42, 43]”

- Can you comment on the number of rescue attempts that were done for each of the mutant viruses? After how many attempts did the authors determined that a virus could not be rescued? How were the rescue attempts controlled? Please also clarify if all viral stocks were sequenced after rescue and prior to passage in eggs, to confirm the sequence integrity of each rescue. Include that information in detail in the methods section.

**Response:** This information is included in the Materials and Methods section of the revised manuscript:

Lines 302-305: “For each mutant, three independent rescue experiments were performed. All our rescue experiments included a positive control that contained wild type (WT) HA and a negative control that contained no HA.”

Lines 305-307: “Viral RNAs were extracted, reverse transcribed by Superscript III reverse transcriptase (Thermo Fisher Scientific), and sequenced by Sanger sequencing to confirm its sequence integrity before passaging in eggs.”

- For the figure legends 1B, 2B and E, and 3B-D, provide more information on the experiment? e.g. since the results of the rescue attempts in the 293T/hMDCK… For expalme in Line 356, “…in a virus rescue experiment in mammalian cells (293T/hMDCK) after XX attempts”. Indicate what the error bars are (e.g. Line 359: SD of three experiments represented by the error bars).

**Response:** In the revised manuscript, the following additional information is provided in the legends of Figures 1C-D, 2B, 2C, 2E, and 3B-D:

“… in a virus rescue experiment in mammalian cells (293T/hMDCK).”

“The means of three independent experiments are shown with SD indicated by the error bars.”

- The use of “egg-adaptation pathway” is a bit confusing, as this would imply a unique way in which the mutations might generate. The data shown (e.g. Figs 2, 3 and 4) indicate that different mutations (amino acid variations) in deed occur depending on the genotype of the initial human H3N2 strain. Hence, it appears that alternative amino acids (e.g. more then one residue) can emerge at different positions as compensatory mutations. Therefore, I suggest revising the use of “egg-adaptation pathway” throughout the manuscript, including the tittle, to better reflect the conclusions of the study. For example, a tittle a long these lines: “Egg-adaptation compensatory mutations of human influenza H3N2 virus are dependent on natural evolution”, might be more aligned with the results shown.

**Response:** Thank you for the suggestion. We have replaced “egg-adaptation pathway” by “egg-adaptive mutation” throughout the manuscript, including the title, to minimize confusion.

**Part III – Minor Issues: Editorial and Data Presentation Modifications**

Reviewer #1:

It might be nice to add details about the new 2022-23 Northern Hemisphere H3N2 vaccine strain (and current SH vaccine) which doesn’t have the N158 glycosylation site.

**Response:** Thank you for the suggestion. The latest seasonal H3N2 vaccine viruses for both the Northern and Southern Hemisphere in 2022-23 season are A/Darwin/9/2021 (egg-based) and A/Darwin/6/2021 (cell-based), which are now included in Figure 1A and Supplementary Table 1 of the revised manuscript.

Reviewer #2:

1. At the end of figure 1 please include a phylogenetic tree of the different viruses being tested and the mutations associated with which. This will help clarify how the egg-adaptation and H3N2 strains relate. Alternatively, a structure of HA and which residues are associated with each strain would be useful.

**Response:** Thank you for your suggestions. we have added a figure (Figure 1B) to summarize the relationship between different egg-adaptive mutations and strains of interest.

2. Line 150, please clarify this is circulating virus and not egg-grown virus.

**Response:** Thank you for pointing out. This sentence is amended in the revised manuscript (lines 167-168):

“T160 and N190 have reached high occurrence frequency in recently circulating human H3N2 strains.”

3. Line 175, can the authors expand on why RBS height may matter? I’d assume this obstructs receptor binding, but it’s not clear.

**Response:** Thank you for your questions. This is clarified in the revised manuscript (lines 191-194):

“As demonstrated in our previous study, G186V significantly increases the RBS height [14]. In contrast, L194P decreases the RBS height. The co-existence of these two mutations with opposite structural effects destabilizes the 190-helix and abolishes receptor binding [14].”

4. Line 215 – should read “which all belong to clade…”

**Response:** Fixed.

5. The authors do a lot of virus rescuing but it’s not explicitly clear how they did so. They seem to use reverse genetics to generate their viruses and to rescue them in MDCK cells and then use eggs to show stability of mutations. Clarity on methods are appreciated.

**Response:** Details of methodology for virus rescue are clarified in the materials and methods section of the revised manuscript (lines 281-307):

“**Virus rescue experiments**

All H3N2 viruses generated in this study were based on the influenza eight-plasmid reverse genetics system [40, 41]. The HA and neuraminidase (NA) genes of the strains of interest (Supplementary Table 2) were synthesized by Sangon Biotech, and cloned into the pHW2000 vector as previously described [14]. For HA, the ectodomain was from the strains of interest, whereas the non-coding region, N-terminal secretion signal, C-terminal transmembrane domain, and cytoplasmic tail were from H1N1 A/PR/8/34 (PR8). For NA, the entire coding region was from the strains of interest, whereas the non-coding region of NA was from PR8. Mutations were introduced into the constructs by polymerase chain reaction (PCR). Primers used in this study were produced by Integrated DNA Technologies. Recombinant chimeric 6:2 reassortant viruses with the six internal genes (PB2, PB1, PA, NP, M, and NS) from PR8 were rescued by transfecting a co-culture of HEK 293T and hMDCK cells at a 6:1 ratio with 70% confluence in 6-well plate. Transfection was performed using 16 μL of TransIT-LT1 (Mirus Bio) and 1 μg each of the eight plasmids encoding the corresponding influenza virus segments (a total amount of 8 μg of DNA). At 6 h post-transfection, medium was replaced with 1 mL of MEM. At 24 h post-transfection, another 1 mL of MEM supplemented with 1 μg mL-1 tosylphenylalanyl chloromethyI ketone (TPCK)-trypsin was added. Supernatant was harvested at 72 h post-transfection, all of which was further inoculated into hMDCK cells at 90% confluence in a T75 flask. Supernatant of the hMDCK cells was harvested when more than 70% of cells had cytopathic effect (CPE). If less than 70% of cells had CPE by 72 h post-infection, cell supernatant was harvested regardless. Virus titer was determined by titration in hMDCK cells as previously described [42, 43]. For each mutant, three independent rescue experiments were performed. All our rescue experiments included a positive control that contained wild type (WT) HA and a negative control that contained no HA. Viral RNAs were extracted, reverse transcribed by Superscript III reverse transcriptase (Thermo Fisher Scientific), and sequenced by Sanger sequencing to confirm its sequence integrity before passaging in eggs.”

Reviewer #3:

Line 95-97) What do authors mean by background mutations? Are these egg mutations having a deleterious impact on the antigenicity of the virus?

**Response:** We agree that the usage of the term “background mutation” is causing unnecessary confusion and therefore have removed them from the revised manuscript. In addition, the antigenic effect of T160K (one of the “background mutations”) was clarified in the revised manuscript (lines 111-113):

“Similarly, all the Sing16 and Switz17 mutants were constructed in the presence of T160K, which was found in all egg-passaged strains derived from Sing16 and Switz17 in GISAID and could alter the antigenicity of the virus (Figure 1B) [5].”

Line 150-151) What do authors mean by “T160, which introduced an N-glycosylation site at N158”? Does this N-glycosylation occur due to a higher accessibility to this site of glycosyltransferases after the T160 mutation takes place?

**Response:** In 2014-2015 influenza season, K160 mutates to T160, which changed the amino acid sequence at residues 158-160 from NFK to NFT. N-glycosylation occurs at the Asn of the motif NXT, where X can be any amino acid except Pro. As a result, the emergence of T160 causes N158 to be glycosylated.

Line 226-227) Do authors mean that if for instance there is a prevalent circulating H3N2 strain but it is likely that when growing it in eggs there appears remarkable antigenic changes, it is better to select another circulating strain, though less prevalent in humans, but less prone to antigenic changes in eggs? Of course we are not considering here the use of mammalian cell lines to grow these viruses, we assume only that the egg platform is the only possibility.

**Response:** The following is our current hypothesis. If a prevalent circulating H3N2 strain (strain X) is prone to egg-adaptive mutation that can significantly alter its antigenicity, it might be better to select a strain (strain B) that is antigenically similar to strain X and yet antigenically stable in eggs. In other words, if the antigenic distance between egg-adapted strain B and wild type strain X is smaller than the antigenic distance between egg-adapted strain X and wild type strain X, we should select strain B instead of strain X as vaccine strain. We are currently trying to follow up on this hypothesis and hope to publish the results as a separate study.

Discussion point) Is the rationale of this study based on the egg-adapted mutations discussed in this study since they are the most prevalent seen in H3N2 strains? Are there other relevant mutations not mentioned in this study?

**Response:** Thank you for your suggestion. Our study is centered around G186V and L194P because they are the most prevalent egg-adaptive mutations as mentioned in the introduction (line 71):

“G186V and L194P are the two most common egg-adaptive mutations in H3N2 HA [14].”

There are indeed other egg-adaptive mutations that were not mentioned in our original manuscript, but are now included in the discussion section of the revised manuscript (lines 223-225):

“Besides L194P, many other egg-adaptive mutations, albeit at a lower prevalence, were observed in historical vaccine strains, such as H156Q, H156R, H183L and A196T [14, 25-27].”

Discussion point) according to the data provided by the authors, it seems the deleterious mutation L194P is less prevalent in egg-passaged H3N2 strains. Do authors believe that mismatches to current circulating H3N2 strains due to egg-passaging are less likely to occur? Discussion on this topic would be interesting for the audience.

**Response:** Thank you for this interesting thought, which is now addressed in the discussion section of the revised manuscript (lines 247-251):

“Currently, 3C.2a1b.2a2 is the dominant clade of circulating human H3N2 virus. Although our data show that egg-adaptive mutation L194P is unlikely to occur in clade 3C.2a1b.2a2, egg-adaptive mutations D186N and D225G are common in this clade [15]. However, the antigenic effects of D186N and D225G in clade 3C.2a1b.2a2 remain to be explored.”

Reviewer #4:

1. In the model on Figure 3, for orientation it would be helpful to include/highlight the locations of residues 160 and 98.

**Response:** Thank you for your suggestion. We have highlighted the location of residues 160 and 98 in Figure 3 of the revised manuscript.

2. In the Figure 4 legend, include the method and bootstrap used to construct the phylogenetic tree and whether the tree was rooted or not.

**Response:** In Figure 4 legend of the revised manuscript (lines 456-458), additional information has been provided:

“A rooted phylogenetic tree was built using maximum likelihood (ML) method with 100 bootstraps on the HA sequences of 61 human H3N2 strains without egg-passaging (unpassaged strains).”

Detailed method has also been described in the materials and methods section of the revised manuscript (lines 266-272):

“Information for the full-length HA sequences of viruses from 2019 to 2021, with and without egg-passaging, is shown in Supplementary Table 4. Sequences were aligned by MAFFT version 7 (https://mafft.cbrc.jp/alignment/software/) with default setting [36]. Passaging history was determined by parsing the regular expression in FASTA headers as described [37]. The frequencies of amino acids were plotted using WEBLOGO (https://weblogo.berkeley.edu/logo.cgi) [38]. Phylogenetic tree was built and rooted using maximum likelihood (ML) method by Geneious Prime (Geneious), with 100 bootstraps.”

3. Line 52; change to “which is the prototypical receptor for avian influenza viruses”

**Response:** This change is made as suggested.

4. Line 52; Change to “Since the HA RBS partial…”

**Response:** The word “the” is added as suggested.

5. Line 62; should be “discovered”

**Response:** Fixed.

6. Line 153; is this supposed to be 2020 and not 2021??

**Response:** Our analysis shows that the frequency of N190 was <50% in 2020 and started to become dominant in 2021 with >90% frequency. So we prefer to use “2021” rather than “2020”.

7. Line 155; it should be “titers”

**Response:** Fixed.

8. Line 157-158; change to “…as determined in the egg-adapted viruses”

**Response:** This sentence is revised accordingly.
